# Ferroptosis as an immunometabolic checkpoint in brain tumours: spatial vulnerabilities and therapeutic opportunities

**DOI:** 10.1186/s13041-026-01344-9

**Published:** 2026-08-24

**Authors:** Maher Nassor, Saim Chaudhry, Fahmida Zahin, Subham Roy, Kay Paulina K. Odei, Princess Afia Nkrumah-Boateng, Andrew Awuah Wireko

**Affiliations:** 1 School of Medicine, University of Sunderland, Sunderland, England; 2 University of Edinburgh Medical School, Edinburgh, UK; 3 University of Buckingham, Buckingham, UK; 4 Hull York Medical School, University of York, York, UK; 5 Independent researcher, Toronto, Canada; 6 University of Ghana Medical School, Accra, Ghana; 7 Inter-Continental Omni-Research in Medicine Collaborative, Berlin, Germany

**Keywords:** Ferroptosis, Brain tumours, Immunometabolism, Spatial heterogeneity, Iron metabolism, Lipid peroxidation, Tumour microenvironment, Therapy resistance, Molecular neuro-oncology

## Abstract

Brain tumours, particularly glioblastoma, remain among the most lethal cancers due to considerable spatial variation, metabolic adaptability, immune suppression, and resistance to standard therapies. Ferroptosis, an iron-dependent form of regulated cell death driven by lipid peroxidation, presents a candidate target for new treatments in these tumours. This review examines ferroptosis as an immunometabolic checkpoint in brain tumours, emphasising how iron metabolism, glutathione depletion, lipid remodelling, and microenvironmental factors influence vulnerability across different regions. Using the SANRA framework, relevant studies from major sources were analysed to compile mechanistic, preclinical, and translational insights across glioblastoma and other brain tumour types. Evidence shows that brain tumours often activate ferroptosis-inhibiting pathways, including GPX4, *SLC7A11*, and *NRF2*, to promote survival, invasion, and therapy resistance. At the same time, recurrent or treatment-persistent tumour states may develop specific weaknesses to ferroptosis, especially in metabolically stressed or dormant cell populations. Ferroptosis interacts with immune responses: CD8 + T-cell-derived IFN-γ can trigger tumour ferroptosis, although this process may also foster immunosuppressive microenvironments depending on the context. Spatial heterogeneity adds complexity, as hypoxic tumour cores, invasive edges, and perivascular areas vary in oxidative stress, lipid metabolism, immune cell presence, and drug access. In conclusion, ferroptosis acts both as a tumour survival mechanism and as a potential vulnerability to target. Progress depends on integrating spatial and single-cell approaches, biomarker-driven patient stratification, and delivery methods that consider the blood-brain barrier to effectively and safely induce ferroptosis. This strategy highlights ferroptosis as a key focus for personalised neuro-oncology and combination therapy development.

## Introduction

Brain tumours comprise a heterogeneous group of neoplasms characterised by the uncontrolled proliferation of abnormal cells within the central nervous system (CNS) [1]. Their global burden has increased substantially in recent decades, with annual incidence rising from approximately 170,000 to over 350,000 cases between 1991 and 2021 [1]. Prognosis varies widely across tumour subtypes; however, outcomes for many primary malignant brain tumours remain poor, with five-year survival rates as low as 7% for glioblastoma and approximately 30% for CNS lymphomas [2, 3]. Despite advances in multimodal treatment strategies, including maximal surgical resection followed by radiotherapy and temozolomide-based chemotherapy as established in the Stupp protocol [4], glioblastoma, the most aggressive primary brain tumour, continues to carry a median survival of only 12–15 months [5, 6].

Given its aggressive biology and extensive characterisation, glioblastoma serves as a valuable model through which broader challenges in brain tumour biology and treatment can be examined. Central to these challenges is the complex and dynamic tumour microenvironment (TME), shaped by interactions between tumour cells, immune populations, and the unique physiological constraints of the CNS. Brain tumours exhibit profound disruption of the blood–brain barrier (BBB) and associated systems such as the glymphatic network, with important implications for both immune regulation and therapeutic delivery. For instance, medulloblastomas can upregulate drug efflux transporters, limiting chemotherapeutic efficacy [7], while glioblastoma remodels both BBB permeability and glymphatic function to establish a highly immunosuppressive, “immune cold” microenvironment [7]. In this state, impaired antigen drainage and the accumulation of immunosuppressive mediators, including TGF-β, IL-10, and myeloid-derived suppressor cells, collectively inhibit effective T cell activation and proliferation [8].

Genetic and epigenetic heterogeneity further complicates therapeutic response across brain tumours. Variations in key molecular determinants, such as isocitrate dehydrogenase (IDH) mutations and O6-methylguanine-DNA methyltransferase (MGMT) promoter methylation, significantly influence treatment sensitivity and clinical outcomes [9]. Temozolomide, the current standard chemotherapeutic agent for glioblastoma, exerts its cytotoxic effects through deoxyribonucleic acid (DNA) alkylation; however, its efficacy is frequently limited by MGMT-mediated DNA repair. Only a minority of patients (~ 30%) exhibit MGMT promoter methylation, resulting in reduced enzyme activity and enhanced treatment sensitivity, while the majority derive limited benefit [10]. Such intertumour variability underscores the need for therapeutic strategies that can overcome or bypass these resistance mechanisms.

At the intratumoural level, spatial heterogeneity represents an additional and critical barrier to effective treatment. Glioblastomas, and other high-grade brain tumours, are organised into distinct ecological niches including hypoxic cores, angiogenic regions, and invasive margins each characterised by unique cellular compositions and functional states [11]. Hypoxic niches, in particular, support glioma stem cells (GSCs), which maintain tumour propagation and therapeutic resistance through hypoxia-inducible factors such as HIF-1 and HIF-2 [12]. Meanwhile, brain tumour–initiating cells can infiltrate beyond the primary tumour boundary, contributing to diffuse invasion and high recurrence rates following surgical resection [13]. These spatially distinct populations collectively drive tumour adaptability and resilience.

Metabolic reprogramming is a unifying feature underpinning both tumour progression and immune evasion in brain tumours. Unlike normal neural tissue, which relies predominantly on oxidative phosphorylation, malignant cells preferentially utilise aerobic glycolysis, even under oxygen-rich conditions. This metabolic shift not only supports rapid proliferation but also creates a resource-competitive environment that deprives infiltrating immune cells of essential nutrients, further reinforcing immunosuppression within the TME [14, 15]. Increasingly, it is recognised that such metabolic adaptations are closely integrated with immune regulatory pathways, suggesting the existence of interconnected immunometabolic checkpoints governing tumour survival.

Ferroptosis, a regulated form of iron-dependent lipid peroxidation–driven cell death, has recently emerged as a key candidate within this framework. Distinct from apoptosis and other canonical cell death pathways, ferroptosis is governed by iron metabolism, redox homeostasis, and lipid peroxidation dynamics, positioning it at the intersection of tumour metabolism and immune regulation. Here, we define an immunometabolic checkpoint as a regulatory node at which a cell’s metabolic state (iron, lipid, and redox handling) determines not only whether that cell undergoes ferroptosis, but also the downstream immune consequence of that decision—whether its death or survival promotes or restrains anti-tumour immunity [119]. Importantly, ferroptosis induction has been shown to promote the release of damage-associated molecular patterns (DAMPs), enhancing antitumour immune responses and T cell activation [8, 16]. Concurrently, increased PD-L1 expression following ferroptotic stress suggests potential synergy with immune checkpoint blockade [17, 18]. Critically, as ferroptosis operates independently of DNA damage repair pathways, it offers a therapeutic strategy that may circumvent resistance mechanisms such as MGMT-mediated repair, addressing a central limitation in current glioblastoma treatment.

Emerging evidence further indicates that susceptibility to ferroptosis is not uniform across tumours but is shaped by spatial and cellular heterogeneity within the TME. Distinct tumour regions may exhibit differential expression of ferroptosis regulators, antioxidant systems, and lipid metabolic pathways, creating discrete and potentially targetable vulnerabilities. Advances in spatial transcriptomics, single-cell sequencing, and integrative multi-omics approaches now enable high-resolution mapping of these heterogeneities, providing new insights into the spatial organisation of ferroptotic sensitivity across brain tumours.

Whereas prior reviews have largely treated ferroptosis in glioma as a single tumour-cell-intrinsic vulnerability, this review adds three elements not jointly addressed elsewhere: an explicit comparison of immunometabolic ferroptosis patterns across glioma, medulloblastoma, meningioma, and brain metastasis; a spatial map linking each tumour niche to its dominant ferroptosis regulators and therapeutic vulnerabilities; and a dedicated assessment of the CNS-specific toxicity and delivery constraints that any ferroptosis-based therapy must overcome. In this context, this review aims to position ferroptosis as an immunometabolic checkpoint in brain tumours, using glioblastoma as a primary model while extending insights across CNS malignancies. We first examine the molecular mechanisms linking ferroptosis with tumour metabolism and immune regulation. We then explore how spatial heterogeneity within the TME shapes ferroptotic vulnerabilities, drawing on recent spatial and multi-omics data. Finally, we discuss current and prospective therapeutic strategies, including ferroptosis induction, combinatorial immunotherapy approaches, and targeted delivery systems, highlighting both opportunities and translational challenges. Through this integrated perspective, the review seeks to provide a framework for exploiting ferroptosis in a spatially and immunologically informed manner to improve outcomes in brain tumours.

## Methodology

This narrative review utilised the Scale for the Assessment of Narrative Review Articles (SANRA) framework to ensure methodological rigour, transparency, and relevance [19].

### Eligibility criteria

A broad spectrum of study designs was considered, including basic science studies, translational preclinical investigations, observational studies, case-control analyses, cohort studies, randomised controlled trials, systematic reviews, and meta-analyses where relevant. Given the evolving nature of the field, high-quality mechanistic and experimental studies were prioritised alongside clinically pertinent literature. Both adult and paediatric brain tumours were included, with paediatric evidence emphasised where available. Only articles published in English were considered, with the search extending from database inception to the date of the final search.

Studies were included if they examined ferroptosis-related pathways in brain tumours, the relationship between ferroptosis and immune or metabolic regulation, or the therapeutic relevance of ferroptosis induction or inhibition within the context of brain tumour biology, treatment response, or spatially distinct tumour niches. Particular attention was given to studies exploring the interface between ferroptosis and the tumour microenvironment, including immune cells, hypoxia, oxidative stress, and regional metabolic adaptations. Conference abstracts lacking full-text articles, non-peer-reviewed work, blog posts, opinion pieces without primary analysis, and preprints were excluded.

### Search strategy

A structured literature search was conducted across major databases, including PubMed/MEDLINE, Scopus, Embase, Web of Science, CINAHL, IEEE Xplore, and the Cochrane Library up to 6 March 2026. Search terms were devised to capture the core concepts of the review, incorporating combinations of keywords and subject headings including: “ferroptosis”, “brain tumours”, “glioma”, “glioblastoma”, “medulloblastoma”, “ependymoma”, “meningioma”, “brain metastasis”, “immunometabolism”, “tumour microenvironment”, “spatial heterogeneity”, “iron metabolism”, “lipid peroxidation”, “glutathione peroxidase 4”, “system X_c_^−^”, “SLC7A11”, “therapy resistance”, “radiotherapy sensitisation”, “immunotherapy”, and “metabolic vulnerabilities”. Boolean operators and Medical Subject Headings (MeSH) were employed as shown by the following:

### Article selection and assessment

Duplicated articles were removed using Endnote 2025. All remaining articles were independently reviewed at the title and abstract level and full-text level by four authors (M.N., S.C., P.A.N.B and A.A.W.) in accordance with the inclusion and exclusion criteria. Any disagreements regarding inclusion of articles in the review were discussed between the supervising author (A.A.W.) and the reviewing authors, with the final decision made by the supervising author. Additionally, a manual search of the reference lists of key reviews and eligible publications was conducted to identify additional relevant articles. Duplicate records were removed using Endnote 2025. The remaining articles were screened at the title/abstract and full-text levels against the inclusion and exclusion criteria above, and those meeting all criteria were retained for narrative synthesis in this review.

## Mechanisms of ferroptosis: fundamentals and key pathways

### Ferroptosis: iron-dependent cell death

The term ferroptosis, first introduced in 2012, describes a form of regulated cell death that is dependent on iron [20, 21]. Regulated cell death is governed by specific molecular mechanisms, distinguishing it from accidental cell death caused by unforeseen mechanical, physical, or chemical stress [22]. Ferroptosis results from alterations in glutathione-dependent lipid oxidation pathways, leading to the accumulation of lipid-based reactive oxygen species (ROS) [23, 24]. This form of cell death is implicated in numerous diseases, including hepatic, renal, and neurological disorders [25].

Ferroptotic cell death is regarded as morphologically, genetically, and biochemically distinct from other forms of regulated cell death such as apoptosis, necrosis, and autophagy [22]. For instance, apoptosis is characterised by activation of caspases; necrosis involves cellular swelling and membrane rupture; autophagy is regulated through lysosomal degradation [22].

### Core molecular regulators

Various molecular regulators are involved in the mechanisms governing ferroptosis. As this cell death modality is dependent on iron, iron metabolism assumes a pivotal role. For example, the expression of transferrin receptor 1 (*TFR1*), which mediates iron ion import into cells, can be suppressed by heat shock protein B1 (HSPB1). Consequently, overexpression of HSPB1 diminishes intracellular iron levels, thereby inhibiting ferroptosis [24]. Likewise, upregulation of cytoplasmic ferritin, the protein responsible for iron storage, can prevent iron overload and ferroptosis [24]. The transcription factor iron response element-binding protein 2 enhances the expression of ferritin-related genes, such as ferritin light chain (*FTL*) and ferritin heavy chain 1 (*FTH1*), which in turn can inhibit erastin-induced ferroptosis [24].

Lipid peroxidation pathways are also central to ferroptosis regulation. Phospholipids containing polyunsaturated fatty acids (PUFAs), particularly the coenzyme-A derivatives arachidonic acid (AA) and adrenic acid, are highly susceptible to oxidation, thus promoting ferroptosis [24]. These PUFA derivatives, also known as oxidised phosphatidylethanolamines, undergo esterification by the enzyme ACSL4, converting AA and adrenic acid into arachidonic acid-CoA and adrenic acid-CoA, respectively [24]. These intermediates are involved in synthesising membrane phospholipids with negative charges. The peroxidation of these PUFA-rich phospholipids by lipoxygenase (LOX) enzymes compromises membrane integrity and permeability, culminating in rupture and cell death [26].

Antioxidant systems are likewise instrumental in controlling ferroptosis, especially in mitigating cellular stress. System X_c_^−^, an antioxidant transporter embedded in the cell membrane, consists of the subunits solute carrier family 7 member 11 (SLC7A11) and solute carrier family 3 member 2 [24]. This transporter, functioning as a cystine/glutamate antiporter, imports cystine and exports glutamate. The imported cystine is reduced to cysteine, which is utilised in synthesising gamma-glutamyl-cysteine-glycine (GSH), an antioxidant tripeptide. GSH serves as a cofactor for glutathione peroxidase 4 (GPX4), which reduces lipid hydroperoxides, thereby preventing their accumulation and conferring resistance to ferroptosis [24, 26].

### Role of ROS, glutathione depletion, and mitochondria in ferroptosis

Ferroptosis is a regulated form of cell death characterised by the iron-dependent accumulation of lipid ROS. Dixon et al. [27] were the first to distinguish it from apoptosis, necrosis, and autophagy. Its progression relies not merely on general oxidative stress but rather on the uncontrolled peroxidation of polyunsaturated phospholipids within cellular membranes. [27] The primary defence mechanism against this process is the system X_c_^−^ glutathione (GSH)–GPX4 axis. System X_c_^−^ facilitates the import of cystine for intracellular cysteine synthesis, which is essential for GSH production; subsequently, GPX4 employs GSH to detoxify lipid hydroperoxides. When cystine uptake is inhibited or GSH levels are depleted, GPX4 activity declines, leading to the accumulation of lethal lipid peroxides and membrane damage characteristic of ferroptosis [23, 28].

Mitochondria also participate in ferroptosis; however, their involvement depends on specific circumstances rather than being universally essential. Experimental evidence suggests that mitochondria are particularly significant in ferroptosis induced by cysteine deprivation, where processes such as the tricarboxylic acid cycle, electron transport chain, and glutaminolysis augment the production of ROS and lipid peroxides [29]. Conversely, certain forms of ferroptosis triggered by GPX4 inhibition can occur with less reliance on mitochondrial functions, indicating that mechanisms vary according to the initial stimulus. Morphologically, ferroptotic cells typically display smaller mitochondria, increased membrane density, and reduced or absent cristae—features that help differentiate ferroptosis from other cell death modalities [30].

In oncology, these mechanisms are of particular importance because tumour cells often encounter elevated oxidative stress while relying on antioxidant defences for survival. This creates a metabolic vulnerability: cancers that depend heavily on *SLC7A11*, *GPX4*, or similar redox buffering pathways may be notably susceptible to therapies that induce ferroptosis, especially in cases where the disease exhibits treatment resistance [28].

### Comparison with other cell death types and cancer biology implications

Ferroptosis fundamentally differs from apoptosis, necroptosis, and other regulated modalities of cell death in its mechanism, morphology, and biochemical signature. Apoptosis is typically caspase-dependent and characterised by chromatin condensation, membrane blebbing, and the formation of apoptotic bodies. In contrast, ferroptosis is characterised by iron-dependent lipid peroxidation, an inability to repair membranes, and notable mitochondrial shrinkage, occurring without the classic nuclear fragmentation seen in apoptosis. Necroptosis, on the other hand, is driven by signalling through *RIPK1*,* RIPK3*, and *MLKL*, while pyroptosis relies on inflammasome activation and membrane pore formation mediated by gasdermin. The Nomenclature Committee on Cell Death recognises ferroptosis as a distinct regulated cell death modality within this broader framework [31].

These distinctions are of considerable importance in cancer biology. Many tumours develop resistance to apoptosis through mutations in p53, BCL-2 family signalling, or caspase-associated pathways, thereby reducing the efficacy of conventional treatments that depend on apoptotic cell death. Ferroptosis presents an alternative vulnerability, as it targets tumour reliance on iron metabolism, lipid metabolism, and antioxidant defence systems rather than apoptotic pathways. This is especially relevant in aggressive and therapy-resistant cancers, where mesenchymal-like or drug-tolerant states may exhibit increased sensitivity to ferroptosis [23, 28]. It is also important to recognise the overlap between different cell death pathways, since regulated networks are interconnected and can influence treatment outcomes in complex ways [31].

In the context of cancer therapeutics, ferroptosis is not merely another cell death pathway but a strategically significant bypass mechanism that could circumvent resistance to apoptosis-based treatments. Its importance becomes even more pronounced in tumours characterised by metabolic rewiring, high iron requirements, and oxidative imbalance, thereby establishing ferroptosis as a compelling target in precision oncology.

## Ferroptosis in brain tumours: pathophysiological context

### Evidence of ferroptosis dysregulation in brain tumours

Ferroptosis dysregulation is observed in brain tumours. For instance, increased expression of *GPX4* is found in glioblastoma cells compared to healthy astrocytes. This plays a role in promoting tumour growth and survival, as *GPX4* overexpression can inhibit ferroptitic cell death in these tumours [32]. High GPX4 levels are also found to be correlated with therapy resistance in patients with Glioblastomas [33].

The xCT pathway also regulates ferroptosis in brain tumours. Studies have found that higher levels of xCT can contribute to growth, survival and invasion of glioma cells through various mechanisms, including the limitation of ROS aggregation [34, 35]. Another study found a strong correlation between temozolomide-resistance in patients with recurrent glioblastoma and the increased expression of xCT [35].

*FSP1* plays a role in ferroptosis resistance. A recent study found increased expression of *FSP1* in patients with recurrent glioblastoma tumours. This suggests that FSP1 plays a role in therapeutic resistance of these tumours [33]. Antioxidant signalling mediated by *NRF2* promotes ferroptosis resistance in several cancers, including glioblastoma [36]. Several ferroptosis regulators are seen in brain tumours, and they all play key roles in the invasion, growth, survival and therapeutic resistance of these tumours.

### Metabolic reprogramming in brain tumours that enhances ferroptosis susceptibility

The dysregulation of iron metabolism plays an important role in ferroptosis susceptibility in brain tumours. Glioblastoma cells in particular show heightened dependency on iron for metabolic functions, termed “ferroaddiction” or “iron addiction” [37]. Glioblastoma cells and GSCs observe upregulation of *TFR1* leading to an influx of iron. These cells also downregulate *FTH1* resulting in reduced iron sequestration. This excess iron triggers lipid peroxidation and ferroptosis [37]. However, glioblastoma cells can develop resistance mechanisms to this ferroptosis susceptibility, including tight regulations of the uptake, export and storage of iron [38, 39].

### Preclinical and clinical observations

Preclinical and clinicopathological investigations increasingly indicate that ferroptosis is not only mechanistically significant in brain tumours but also observable in patient tissue samples and during disease progression. In glioblastoma, immunohistochemical analysis of matched primary and recurrent tumours revealed that relapsed tumours exhibited decreased *GPX4* expression alongside elevated *ACSL4* levels, a pattern consistent with heightened susceptibility to ferroptosis; additionally, increased *ALDH1A3* expression at relapse was identified, suggesting a link to therapy-resistant tumour states [33]. These findings are noteworthy because *GPX4* functions as a vital anti-ferroptotic enzyme, while *ACSL4* facilitates the incorporation of polyunsaturated fatty acids into membrane phospholipids, thereby promoting lipid peroxidation and the execution of ferroptosis. Collectively, this suggests that recurrent Glioblastoma may acquire a biochemical profile that paradoxically renders it more vulnerable to ferroptosis despite its resistance to standard therapies.

Further evidence from tumour samples supports a correlation between markers of ferroptosis resistance and aggressive tumour behaviour. For example, OTUB1 has been reported to be upregulated in glioma tissues, to show a positive correlation with *SLC7A11* expression in clinical samples, and to be associated with poorer overall survival. Mechanistically, OTUB1 stabilises *SLC7A11* and suppresses ferroptosis, thereby supporting glioma stemness [40]. This is pertinent to tumour progression because *SLC7A11* enhances cystine uptake and glutathione synthesis, aiding tumour cells in buffering oxidative stress and avoiding ferroptotic death. Consequently, high levels of anti-ferroptotic proteins in tumour specimens may signify a more aggressive, stem-like, and treatment-resistant phenotype.

Recent preclinical research has also refined this understanding by demonstrating that ferroptosis susceptibility may vary within heterogeneous glioblastomas, rather than being uniform across tumour cells. Banu et al. [41] showed that quiescent, astrocyte-like glioma cell populations exhibit increased mitochondrial ROS production, lipid peroxidation, and glutathione depletion, rendering them particularly sensitive to *GPX4* inhibition. Importantly, treatment of patient-derived early-passage cell lines and human glioma slice cultures obtained during surgery preferentially depleted these quiescent cells [41]. Since these astrocyte-like, slow-cycling tumour cells are more prevalent in recurrent Glioblastoma and are thought to contribute to disease persistence following chemoradiotherapy, this finding provides a compelling rationale for considering ferroptosis as a therapeutic strategy in relapse and minimal residual disease.

**Fig. 1:**
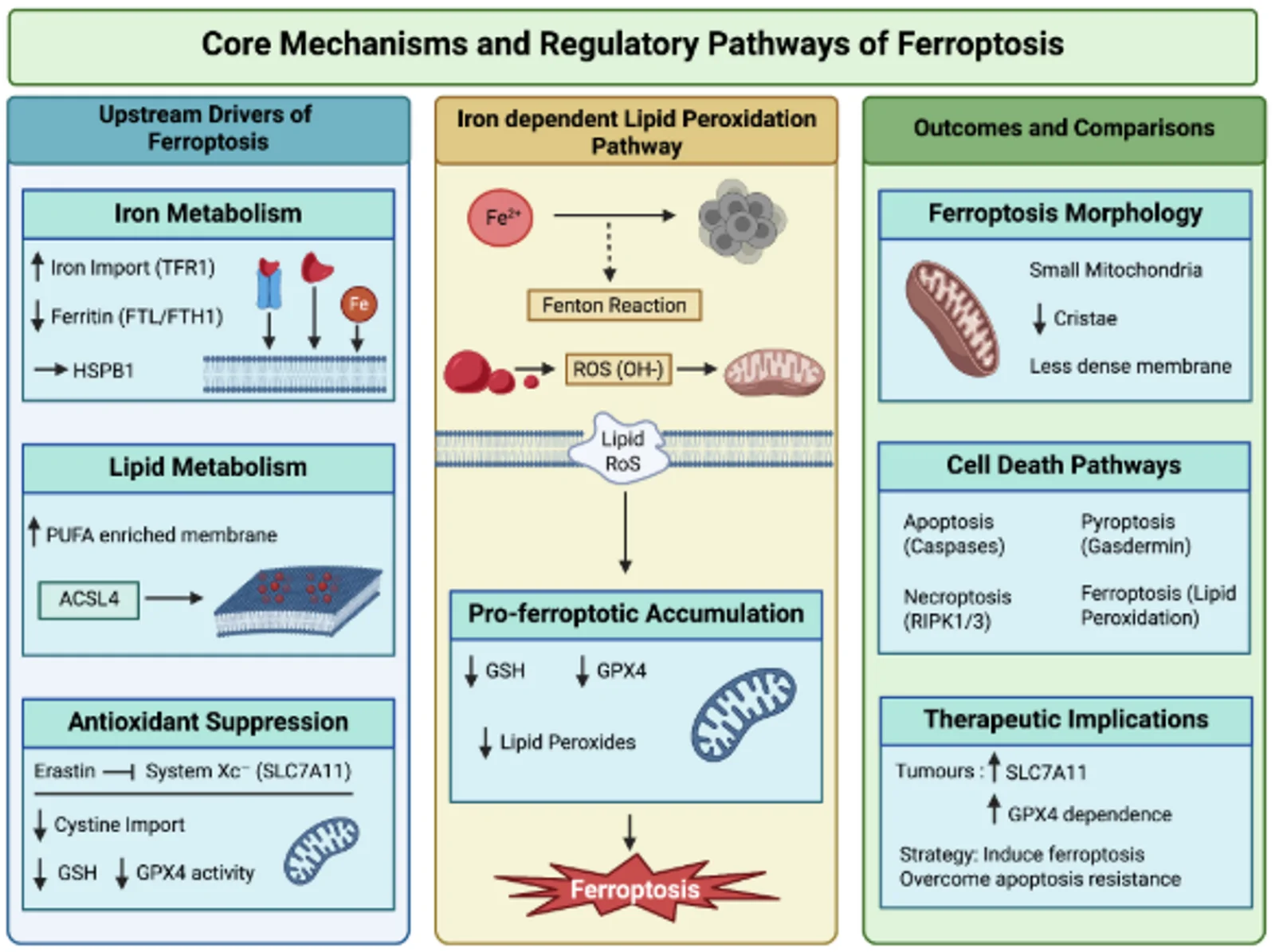
Core mechanisms and regulatory pathways of ferroptosis. Schematic overview of ferroptosis illustrating upstream drivers, including dysregulated iron metabolism, PUFA-enriched lipid membranes, and suppression of antioxidant systems (system X_c_^−^–GSH–GPX4 axis). These converge on iron-dependent lipid peroxidation via the Fenton reaction. This leads to lipid ROS accumulation and ferroptotic cell death. The key morphological features and comparisons with other regulated cell death pathways are shown, alongside therapeutic implications targeting SLC7A11 and GPX4

In summary, the available evidence outlines three key points: firstly, ferroptosis-related markers are detectable in human brain tumour specimens; secondly, recurrent tumours may exhibit increased ferroptosis susceptibility, particularly through decreased *GPX4* and elevated *ACSL4*; and thirdly, the suppression of ferroptosis is associated with tumour progression, stemness, and resistance to treatment, mediated by regulators such as *SLC7A11*. These observations support the notion that ferroptosis is not merely a phenomenon observed in laboratory settings but rather a biologically and clinically relevant process that may elucidate tumour progression and unveil new therapeutic opportunities in aggressive brain tumours. The mechanisms of ferroptosis along with the clinical context have been summarized effectively in Fig. 1.

## Ferroptosis as an immunometabolic checkpoint

### Intersection of ferroptosis with tumour metabolism and immunity

Ferroptosis is increasingly recognised as an immunometabolic checkpoint situated at the crossroads of tumour metabolism, anti-tumour immunity, and redox homeostasis, in addition to being a regulated form of iron-dependent cell death [42]. Mechanistically, ferroptosis results from the accumulation of phospholipid peroxides under conditions where cellular antioxidant systems, particularly the system X_c_^−^–glutathione–GPX4 axis are overwhelmed or suppressed. Its susceptibility is closely controlled by intracellular iron levels, polyunsaturated fatty acids (PUFA)-containing membrane phospholipids, and lipid remodelling enzymes such as ACSL4. Furthermore, the capacity of tumour cells to buffer oxidative stress also influences susceptibility. These processes are modulated by nutrient deprivation, hypoxia, cytokine signalling, and metabolic competition within the TME. Consequently, ferroptosis functions as a metabolic vulnerability intricately linked to immune responses, serving as a decision point through which immune and non-immune signals determine tumour cell vulnerability or survival [43].

This perspective is particularly significant in cancer, where tumour cells often reprogramme cystine uptake, glutamine metabolism, lipid biosynthesis, and iron handling. These adaptations sustain proliferation while resisting oxidative damage. Elevated expression of *SLC7A11* and dependence on *GPX4* enable malignant cells to withstand inflammatory and metabolic stress that would otherwise induce ferroptosis [44]. Therefore, resistance to ferroptosis is not merely a consequence of malignant metabolism but a strategic adaptation supporting immune evasion, therapy resistance, and survival within hostile metabolic environments. Recent reviews have positioned ferroptosis as a central nexus linking cellular metabolism with the immune ecology of the TME, rather than viewing it as an isolated cell death mechanism [45].

Conceptualising ferroptosis as a checkpoint clarifies how the TME can either remain immunologically inert or become permissive to tumour eradication. This depends on whether oxidative and lipid stress are effectively neutralised [46]. Immune-derived stress may be mitigated without triggering significant cell death if tumour cells maintain cystine uptake, glutathione synthesis, and phospholipid redox homeostasis. Conversely, failure of these buffering systems can convert inflammatory and metabolic pressures into ferroptotic damage, potentially reshaping local immune responses [47]. Ferroptosis thus occupies a pivotal position between tumour metabolic adaptation and immune-mediated elimination [48].

### Crosstalk with immune cells

The strongest mechanistic evidence supporting ferroptosis as an immunometabolic checkpoint arises from its interaction with CD8⁺ T cells. In a landmark study, Wang et al. [44] demonstrated that immunotherapy-activated CD8⁺ T cells induce tumour ferroptosis via IFN-γ, which suppresses the expression of system X_c_^−^ subunits *SLC3A2* and *SLC7A1*1 in tumour cells. This results in reduced cystine uptake and glutathione antioxidant defence, thereby increasing lipid peroxidation. This study is pivotal in establishing the connection between the effector function of the adaptive immune response and ferroptosis.

Liao et al. [45] adapted this model to show how IFN-γ collaborates with tumour lipid metabolism to mediate immunogenic tumour ferroptosis through ACSL4. The incorporation of arachidonic acid into membrane phospholipids via ACSL4 is essential for ferroptosis, making tumour lipid metabolism a critical factor in the process. It is important to note that the effectiveness of the immune response depends not only on the presence of cytotoxic lymphocytes but also on the tumour microarchitecture.

Ferroptotic tumour cells may also influence immunity through the release of DAMPs and other immune-stimulating signals. Several reviews and experimental studies suggest that early ferroptotic cells can release molecules such as ATP and HMGB1, which can affect dendritic cell maturation, antigen presentation, and downstream adaptive responses [48]. This has led to the proposal that, under certain conditions, ferroptosis may act as a form of immunogenic regulated cell death, linking tumour metabolic collapse to increased tumour visibility [49, 50]. Nonetheless, this remains an area of ongoing debate, with the literature supporting a more nuanced view of ferroptosis as immunostimulatory only in specific contexts. It is not inherently or uniformly so [47].

Consequently, the most accurate interpretation is that ferroptosis constitutes part of a dynamic feedback loop between tumour and immune cells. Activated CD8^+^ T cells can induce ferroptotic stress in tumour cells, which in turn may modulate the function of antigen-presenting cells and influence subsequent adaptive responses, depending on the circumstances. This underscores the significance of ferroptosis as an immunometabolic checkpoint, rather than merely a mode of cell death [46, 51].

Importantly, however, this feedback mechanism has been primarily characterised in peripheral, immunologically active tumour models, including the ovarian and fibrosarcoma systems in which the CD8⁺ T cell–IFN-γ–system X_c_^−^ axis was initially identified, rather than in cerebral neoplasms [44, 45]. Its relevance to glioblastoma cannot be presumed due to the significantly T-cell-scarce microenvironment of glioblastoma: mature T cells are often excluded, rendered dysfunctional, or sequestered within the bone marrow, thereby depleting the effector population thought to induce immunogenic ferroptosis [125]. Consequently, findings from extracranial, T-cell-replete tumours should only be extrapolated to the central nervous system with explicit caution.

### Dual role in immunosuppression

A notable strength of the current literature is the evolving understanding that ferroptosis is not uniformly advantageous for anti-tumour immune responses. Instead, mounting evidence indicates that the immune effects of ferroptosis depend on the specific context [51, 52]. While ferroptotic death of tumour cells may enhance their destruction and, in some models, promote inflammatory immune responses, other studies suggest that ferroptotic cancer cells can inhibit dendritic cell activation and the adaptive immune response. Of particular relevance is the finding by Wiernicki et al. [45, 46, 50] that ferroptosis in dying cancer cells can impair dendritic cell maturation and the subsequent immune response.

This duality becomes even more significant when considering immune cells, which show considerable variation in their sensitivity to ferroptosis depending on lipid content, antioxidant capacity, and metabolic state [54, 55]. Cytotoxic T cells may induce ferroptosis in cancer cells, yet excessive lipid uptake and peroxidation can also impair ferroptosis in T cells, weakening their anti-tumour activity [56]. Recent reviews highlight the importance of examining ferroptosis within the TME as a whole, rather than focusing solely on tumour cells. This perspective introduces the concept of immune checkpoints, whereby ferroptosis may either promote or hinder immune responses based on the affected cell type and stage of immunity [46, 47, 54, 57].

From the tumour’s perspective, resistance to ferroptosis may contribute to maintaining a “cold” TME. Tumours heavily dependent on the xCT/SLC7A11-GPX4 antioxidant system are more likely to withstand oxidative stress mediated by interferon-gamma (IFN-γ), thus surviving in an inflammatory environment [27, 58]. Ferroptosis resistance may also correlate with immune-suppressive phenotypes, such as dysfunctional antigen presentation, T-cell exhaustion, and myeloid-dominant suppressive microenvironments [59]. Conversely, inducing ferroptosis presents a potential strategy to transform a “cold” tumour into a more “inflamed” or “hot” state by weakening antioxidant defenses and increasing vulnerability to immune attack. The challenge remains to induce ferroptosis selectively, without impairing the anti-tumour immune response [44, 53, 54, 60].

In summary, the literature supports viewing ferroptosis as a regulator of immune quality within the TME, influencing whether oxidative stress and lipid peroxidation foster immunosuppression conducive to tumour survival [46].

### Implications for brain tumours

These issues are especially pertinent in brain tumour biology, as ferroptosis and immune interactions occur within a uniquely characterised anatomical and immunological environment. Gliomas, particularly glioblastoma, develop in a TME marked by severe immunosuppression, abundant microglia and macrophages, limited T-cell infiltration, resistant tumour states, and high metabolic adaptability [61, 62]. Moreover, the BBB hinders drug delivery and the pharmacological induction of ferroptosis in infiltrating tumour tissues. This renders ferroptosis a particularly attractive yet challenging therapeutic approach within neuro-oncology [63].

Recent studies have increasingly highlighted the link between ferroptosis sensitivity, tumour cell demise, and the immune landscape of the glioma microenvironment. This emphasis on myeloid biology is crucial rather than incidental. In glioblastoma, microglia and bone-marrow-derived macrophages collectively make up approximately half of the tumour mass and constitute the primary immune compartment, with their composition and activation status varying according to region and disease stage [62, 126]. In such a myeloid-dominant, T-cell-scarce environment, the immunological consequences of ferroptosis are more likely to be influenced by the lipid metabolism and redox state of tumour-associated macrophages and microglia, as well as by the unique neuroinflammatory characteristics of CNS immunity, rather than by the cytotoxic-lymphocyte circuits that are predominant in peripheral models. Consequently, research has focused on investigating ferroptosis in relation to tumour-associated macrophages and microglia, cytokine signalling, and the redox activity inherent to the glioma microenvironment [64]. Specific research has examined the connection between ferroptosis and glioblastoma, suggesting that targeting this process therapeutically could address limitations of traditional treatments such as chemotherapy and radiotherapy [65, 66].

Caution is essential when translating these findings into clinical practices. The brain TME is not merely a scaled-down version of peripheral tumours but a complex and highly regulated immunological setting. The interactions among tumour cell ferroptosis, myeloid responses, inflammatory damage, and drug delivery across the BBB may be particularly significant in central nervous system tumours [61, 67]. The checkpoint model remains especially pertinent. Ferroptosis could be pivotal in converting oxidative and immune stress into tumour control. Conversely, indiscriminate induction of ferroptosis risks impairing beneficial immune responses or exacerbating pathological inflammation within the brain TME [57, 64, 65].

### Comparative immunometabolic patterns across brain tumour entities

Currently, the strongest evidence exists for glioma and glioblastoma. Compared to other brain tumour types, glioma has the most comprehensive literature on the relationship between ferroptosis, tumour metabolism, treatment resistance, and the immune microenvironment. Consequently, glioblastoma serves as the primary model for exploring ferroptosis as an immunometabolic checkpoint [64, 65, 66].

In contrast, evidence for medulloblastoma remains limited and less immunologically mature. Recent studies suggest that ferroptosis-related transcriptional signatures may vary across molecular subgroups and hold prognostic potential. Additionally, some research indicates that ferroptosis regulation influences radiosensitivity in certain medulloblastoma subtypes, such as the SHH subtype. These findings support the biological and therapeutic relevance of ferroptosis in medulloblastoma [68, 69].

The literature on meningioma is comparatively smaller but suggests mechanistic involvement of ferroptosis. Data imply that sensitivity to ferroptosis could be affected by transcriptional and hormonal signalling pathways, including MEF2C, NF2, E-cadherin, and the PR/p53/HO1/GPX4 axes. However, most evidence centres on tumour cells, with limited information on ferroptosis-immune interactions in meningioma compared to glioma [70, 71, 72].

Evidence concerning brain metastases remains preliminary. Initial findings indicate that ferroptosis-regulating pathways may influence the survival of metastatic cells within the brain microenvironment. Nonetheless, the body of literature is too limited to draw conclusions akin to those in glioblastoma. The pattern across studies shows a gradient: substantial and integrated evidence in glioma, subtype-specific insights in medulloblastoma, mechanistic data in meningioma, and preliminary findings in brain metastases [73].

Overall, this comparison highlights a significant research necessity. Future investigations should move beyond identifying ferroptosis markers to explore how susceptibility correlates with immune composition, treatment response, and the unique challenges of CNS drug delivery and immunity. Developing this immunometabolic framework is important for integrating ferroptosis-based therapies into brain tumour treatment, extending beyond glioblastomas.

A significant proportion of mechanistic evidence originates from cancer cell lines, xenograft models, and ex vivo slice cultures, all of which have inherent constraints that may undermine definitive inference of ferroptosis in human tumours. Cell lines lack the cellular heterogeneity, stromal interactions, vascularisation, and immune complexity characteristic of native tumour microenvironments, potentially exaggerating ferroptosis susceptibility under experimental conditions. Xenograft models provide greater physiological relevance but frequently employ immunodeficient hosts, limiting assessment of immune-mediated ferroptosis and reducing their ability to recapitulate authentic tumour–immune interactions. Similarly, slice cultures preserve tissue architecture only transiently and cannot fully reproduce dynamic immune-cell trafficking, cytokine gradients, vascular perfusion, or long-term metabolic competition present in vivo.

Interpretation of transcriptomic studies also warrants caution. Many ferroptosis-associated signatures are derived from mRNA expression profiles and therefore function as indirect surrogate markers rather than direct measurements of ferroptotic activity. These signatures cannot capture the defining biochemical hallmarks of ferroptosis, including phospholipid lipid peroxidation, iron-dependent membrane damage, or execution of cell death itself. Furthermore, transcriptomic approaches do not account for key post-translational determinants of ferroptosis sensitivity, including GPX4 enzymatic activity, protein stability, enzyme localisation, iron availability, and lipid-remodelling dynamics. Retrospective clinical datasets provide valuable correlative evidence but are similarly limited by patient heterogeneity, treatment-related confounding, and the inability to establish causality. Consequently, associations between ferroptosis-related gene signatures and clinical outcomes should be interpreted as indicative rather than definitive evidence of ferroptotic cell death. Future studies integrating direct biochemical measurements, functional validation, and prospective clinical investigation will be essential to establish the precise role of ferroptosis within tumour immunity and the tumour microenvironment.

## Spatial vulnerabilities in brain tumours

### Spatial heterogeneity in brain tumours

Glioblastoma exhibits considerable spatial and cellular heterogeneity, which contributes to its aggressive nature, resistance to therapy, and poor prognosis. Recent developments in single-cell RNA sequencing (scRNA-seq) and spatial transcriptomics have facilitated detailed mapping of tumour architecture, showing that glioblastoma comprises multiple transcriptionally distinct tumour cell states in specific microenvironmental niches [55, 74, 75]. These studies reveal that tumour cells adopt various phenotypic programmes, resembling neural progenitor-like, astrocyte-like, oligodendrocyte precursor-like, and mesenchymal states, each with distinct metabolic and stress response profiles [55, 75].

Spatial transcriptomic analyses reveal that tumour cell states segregate into spatially organised ecological niches within the tumour mass. For instance, hypoxic and necrotic regions often contain mesenchymal-like tumour cells with immunosuppressive phenotypes, while invasive margins tend to host progenitor-like tumour cells with heightened migratory and proliferative abilities [74, 76]. Imaging-based spatial mapping and radiomic habitat analysis similarly identify distinct tumour habitats characterised by variations in perfusion, metabolism, and vascularisation, supporting the concept of spatially structured tumour ecosystems [77, 78].

Within these heterogeneous tumour habitats, ferroptosis, an iron-dependent form of regulated cell death driven by lipid peroxidation, emerges as a key metabolic vulnerability [38, 79]. Ferroptosis occurs when intracellular iron accumulation and ROS promote lipid peroxidation beyond the capacity of antioxidant systems [39, 80]. Importantly, different tumour cell states display distinct metabolic programmes regulating iron metabolism, lipid synthesis, and antioxidant defences, implying that ferroptosis sensitivity varies spatially within glioblastoma [39, 81].

For example, mesenchymal tumour states often show increased antioxidant capacity and resistance to ferroptosis through upregulation of detoxification pathways [82]. Conversely, certain proliferative or progenitor-like tumour cells possess metabolic features that predispose them to lipid peroxidation and ferroptotic cell death [39]. These spatial vulnerabilities underline the importance of integrating spatial omics techniques with ferroptosis biology to understand tumour ecosystem dynamics.

Three recurrent spatial niches organise these vulnerabilities: hypoxic and necrotic cores, invasive margins, and perivascular niches. Each niche is dominated by a distinct tumour cell state, each with its own set of ferroptosis regulators and, consequently, unique therapeutic susceptibilities. Mapping these regulators onto the tumour’s spatial architecture clarifies why ferroptosis sensitivity varies regionally rather than uniformly, and highlights the region-specific vulnerabilities summarised in the following section (Table 1).

**Table 1:** Spatial niches and their dominant tumour cell state, the principal ferroptosis regulators that set its redox balance, the resulting ferroptosis status, and the therapeutic vulnerability exposed

Spatial niche	Dominant cell state	Key ferroptosis regulators	Ferroptosis status	Therapeutic vulnerability
Hypoxic core	Mesenchymal-like	NRF2-driven antioxidant programme; ROS-detoxifying enzymes; upregulated glutathione/detoxification pathways [82, 88]	Relatively resistant	NRF2/antioxidant-pathway inhibition; Fenton-driven peroxidation from haemorrhagic iron [26, 88]
Invasive margin	Progenitor-like (neural progenitor-/OPC-like)	Lipid-peroxidation-prone metabolism; limited antioxidant buffering [39]	Intrinsically sensitive	ROS-generating and lipid-metabolism-disrupting ferroptosis inducers [26, 66]
Perivascular niche	Glioma stem cells (GSCs)	GPX4 dependence; elevated iron uptake and storage [41, 84]	GPX4-dependent (vulnerable on GPX4 loss)	GPX4 inhibition; exploitation of iron-mediated lipid peroxidation [41, 37]

### Cell state-specific vulnerabilities

Within the heterogeneous tumour populations of glioblastoma, GSCs constitute a crucial subpopulation responsible for tumour initiation, recurrence, and resistance to therapy. These cells typically reside in specialised microenvironments such as hypoxic or perivascular niches and display notable metabolic plasticity [83]. Emerging evidence indicates that these stem-like tumour cells possess distinct metabolic dependencies, making them particularly vulnerable to ferroptosis under certain conditions.

Ferroptosis is closely regulated by cellular antioxidant systems, especially the glutathione-dependent enzyme GPX4, which neutralises lipid peroxides and shields cellular membranes from oxidative damage [38, 80]. Several studies have shown that specific glioblastoma cell states are highly reliant on GPX4 activity for survival, creating a vulnerability to ferroptosis induction that is specific to particular metabolic configurations [41]. Metabolic profiling further reveals that disrupting GPX4 activity selectively triggers ferroptotic death in cellular states characterised by lipid metabolic reprogramming [41].

Iron metabolism also plays a vital role in determining susceptibility to ferroptosis. Cancer stem cells often show increased iron uptake and storage to support their elevated metabolic needs and proliferation [84]. Nonetheless, this iron dependency might paradoxically predispose them to ferroptosis via iron-mediated lipid peroxidation reactions [37]. Studies have demonstrated that hematopoietic stem cells and other progenitor populations exhibit similar vulnerabilities owing to their reliance on iron-dependent metabolic pathways [85].

Various therapeutic approaches aim to exploit ferroptosis sensitivity in glioblastoma. Agents that increase ROS production, inhibit antioxidant pathways, or disrupt lipid metabolism can induce ferroptotic cell death in tumour cells [26, 66]. Experimental research also shows that small-molecule ferroptosis inducers, including disulfiram and natural compounds such as capsaicin, can generate oxidative stress and trigger ferroptosis in glioblastoma cell lines through GPX4-dependent mechanisms [86, 87]. Targeting vulnerabilities specific to cell states thus represents a viable avenue for eradicating therapy-resistant tumour populations.

### Microenvironmental dynamics

The TME is central to regulating ferroptosis sensitivity in glioblastoma. The spatial arrangement of tumour regions creates heterogeneous distributions of oxygen, nutrients, immune cells, and metabolic stressors, all of which influence ferroptotic signalling pathways [83]. Hypoxic and necrotic tumour cores represent prominent spatial niches within glioblastoma. These areas are characterised by severe metabolic stress, elevated ROS levels, and immunosuppressive signalling that collectively promote the development of mesenchymal tumour phenotypes [74]. Nonetheless, tumour cells in these regions often adapt antioxidant responses that reduce their vulnerability to ferroptosis. Activation of *NRF2*-mediated antioxidant pathways and increased expression of ROS-detoxifying enzymes can bolster tumour resistance to oxidative stress [88]. Iron metabolism within the microenvironment further impacts ferroptotic signalling. Iron released from haemorrhagic tumour regions or damaged cells can catalyse lipid peroxidation through Fenton chemistry, heightening ferroptotic stress [26]. Additionally, metabolic interactions between tumour cells and stromal components, including macrophages, endothelial cells, and extracellular matrix elements, modulate oxidative stress and lipid metabolism within the tumour ecosystem [89]. Recent research emphasises the importance of lipid metabolism in ferroptosis regulation. The accumulation of lipid droplets, fatty acid metabolism, and membrane lipid composition influences vulnerability to lipid peroxidation and ferroptotic cell death [90]. Moreover, oxidative lipid signalling involving reactive aldehydes such as HNE and MDA contributes to tumour plasticity and resistance to ferroptosis [91]. Collectively, these findings demonstrate that the TME creates spatial gradients of oxidative stress, lipid metabolism, and iron availability that determine local ferroptosis thresholds.

### Relapse and metastasis contexts

Tumour recurrence is a characteristic feature of glioblastoma and remains a significant obstacle to effective therapy. Recurrent tumours often display substantial molecular and metabolic reprogramming driven by selection pressures induced by treatment. Increasing evidence indicates that ferroptosis-related pathways are integral to these adaptive mechanisms.

Genomic and transcriptomic analyses have identified ferroptosis-associated gene signatures linked to tumour progression and patient prognosis in glioma [92, 93]. In recurrent tumours, changes in lipid metabolism, iron regulation, and antioxidant pathways may promote tumour survival amid oxidative stress [94]. Simultaneously, these metabolic adaptations can create new vulnerabilities to ferroptosis that could be exploited therapeutically.

Bibliometric and systems-level reviews of ferroptosis research in glioma demonstrate a rapidly growing interest in the role of ferroptosis in tumour progression, resistance to treatment, and recurrence [95]. For example, tumour cell populations resistant to conventional therapies might display increased reliance on redox homeostasis pathways, making them more susceptible to ferroptosis induction.

Recent mechanistic investigations have identified specific molecular regulators influencing ferroptosis resistance in glioblastoma. For example, lipocalin-2 signalling via AXL has been shown to confer resistance to ferroptosis in tumour cells [96]. Likewise, adaptive metabolic changes and ferroptosis defence mechanisms may develop during tumour evolution, underscoring the dynamic nature of ferroptosis susceptibility in recurrent disease [81].

These findings imply that spatial mapping of ferroptosis-related pathways within recurrent tumours could identify actionable therapeutic targets and support the development of precision treatment approaches.

### Clinical implications of spatial ferroptotic heterogeneity

Recognising the spatial heterogeneity of ferroptosis holds significant implications for clinical management of glioblastoma. Conventional treatments often regard tumours as uniform entities; however, spatial omics techniques reveal that different tumour regions possess distinct metabolic states and therapeutic susceptibilities [55, 75]. A key implication is the potential for therapies guided by spatial information. By pinpointing tumour regions more vulnerable to ferroptosis, clinicians could target resistant cell populations more effectively. Biomarkers and gene signatures associated with ferroptosis have already demonstrated promise in predicting tumour prognosis and informing targeted interventions [92, 93].

Combination therapies that incorporate ferroptosis induction alongside immunotherapy or metabolic targeting strategies are increasingly being explored. Ferroptosis has been suggested as an immunometabolic checkpoint capable of modifying the TME and boosting anti-tumour immune responses [26, 37]. Nanotechnology-based methods designed to regulate tumour redox balance and metal-dependent cell death pathways may further improve the spatial precision of ferroptosis-targeted treatments [96]. Furthermore, novel approaches aimed at targeting tumour metabolism and microenvironmental interactions could enhance ferroptosis induction. Biomaterial platforms that modulate the TME and promote immune infiltration offer potential strategies to overcome ferroptosis resistance [89]. These techniques could be particularly effective when combined with agents that weaken antioxidant defences or elevate lipid peroxidation within tumour cells. Advances in spatial omics, metabolic imaging, and molecular profiling may also support the development of clinically relevant biomarkers for identifying tumours vulnerable to ferroptosis. Integrating these technologies into clinical practice may facilitate personalised therapies that exploit ferroptosis susceptibilities within heterogeneous tumour ecosystems. Overall, ferroptosis constitutes a crucial immunometabolic checkpoint within the complex spatial organisation of glioblastoma. Understanding how ferroptotic susceptibility varies across tumour cell states and microenvironmental niches might open new pathways for precise treatment, especially in cases of treatment-resistant and recurrent disease.

## Therapeutic targeting with challenges

### Ferroptosis inducers (FINs) as standalone or combination therapies

The ferroptosis inducer (FIN) RAS-selective lethal 3 (RSL3) is a small molecule targeting GPX4 and the NF-κB pathway, inducing ferroptosis in glioma cells. However, a recent study employing glioblastoma cell lines and murine xenograft tumour models demonstrated that downregulation of *GPX4* alone by RSL3 does not effectively induce ferroptosis. Instead, activating the NF-κB pathway in conjunction with *GPX4* suppression triggers ferroptosis and impedes glioma growth and recurrence [97]. A subsequent investigation using glioblastoma cell lines revealed that ferroptosis-inducing agent 56 (FIN56) facilitates ferroptosis through degradation of GPX4 [98]. Erastin, another FIN, inhibits the SLC7A11/xCT transporter, reducing intracellular GSH levels. This decrease compromises the cell’s capacity to neutralise ROS, thereby elevating oxidative stress [99, 100]. Recent evidence also indicates that iron nanoparticles can induce ferroptosis in glioma cells, with enhanced efficacy when combined with therapies such as cisplatin and small interfering RNA (siRNA) [101, 102]. Additionally, certain natural compounds exhibit anti-cancer properties and induce ferroptosis. For example, the curcumin analogue ALZ003 has been shown to inhibit temozolomide-resistant glioblastoma while also promoting ferroptosis by reducing GPX4 expression [T.-C. Chen, [103].

### Synergies with standard treatments

Temozolomide is known to induce apoptosis; however, recent evidence indicates it can also promote ferroptosis by upregulating divalent metal transporter 1 (DMT1), facilitating the transport of iron ions across the cell membrane [104]. Targeting various regulators of ferroptosis may synergistically enhance the sensitivity of glioblastoma cells to temozolomide. For example, a study involving glioma cell lines and rat models demonstrated that *SLC7A11* inhibitors such as erastin and sulfasalazine can augment the anti-tumour effects of temozolomide in glioblastoma cells [105].

The efficacy of radiation therapy, a standard treatment for glioblastomas, can be enhanced through the synergistic effects of ferroptosis inducers. A study using human glioma slice cultures demonstrated that imidazole ketone erastin (IKE), an SLC7A11 inhibitor, combined with radiation therapy, resulted in increased ROS accumulation compared to radiation therapy alone. Similar synergistic effects were observed with the combination of RSL3 and radiation therapy [106].

Induction of ferroptosis may synergise with anti-PD-1/PD-L1 immunotherapy. The TYR03 signalling pathway upregulates the gene SLC3A2, which inhibits tumourigenic ferroptosis. Research using murine models demonstrated that inhibition of TYR03 can sensitise tumours to anti-PD-1 therapy whilst promoting ferroptosis [107].

### Novel strategies

Nanomedicine pertains to the innovative application of nanotechnology in drug delivery, especially important for brain tumours. Ferroptosis nano drugs can traverse the BBB more effectively owing to their smaller size [108, 109]. These nanoparticles also enable more precise targeting of cancer cells while minimising potential harm to surrounding healthy tissue [110]. Recent mouse studies have demonstrated a reduction in glioblastoma tumour sizes following nanoparticle-induced ferroptosis therapies [102, 111]. Nanomedicine presents a promising approach for more efficient delivery of therapies for brain tumours.

Ferroptosis suppressor protein (FSP1) is a glutathione-independent antioxidant that prevents ferroptosis by mitigating oxidative damage, thereby enhancing glioblastoma survival. Pharmacological inhibition with iFSP1 increases lipid peroxidation and sensitises glioblastoma cells to ferroptosis inducers [112].

### Clinical translation

Current research explores the clinical application of these potential therapeutics, with numerous ongoing trials assessing the effectiveness of ferroptosis-inducing agents combined with immunotherapies [113]. Challenges remain in translating ferroptosis-based treatments into clinical practice, notably due to the heterogeneity in ferroptosis-related gene expression across glioblastoma subtypes, which complicates the identification of reliable biomarkers for tumour susceptibility [37]. A recent prognostic study proposed nine long non-coding RNAs as candidate ferroptosis-related biomarkers, aiding in survival prediction and the guidance of immunotherapeutic approaches for gliomas [110].

**Fig. 2:**
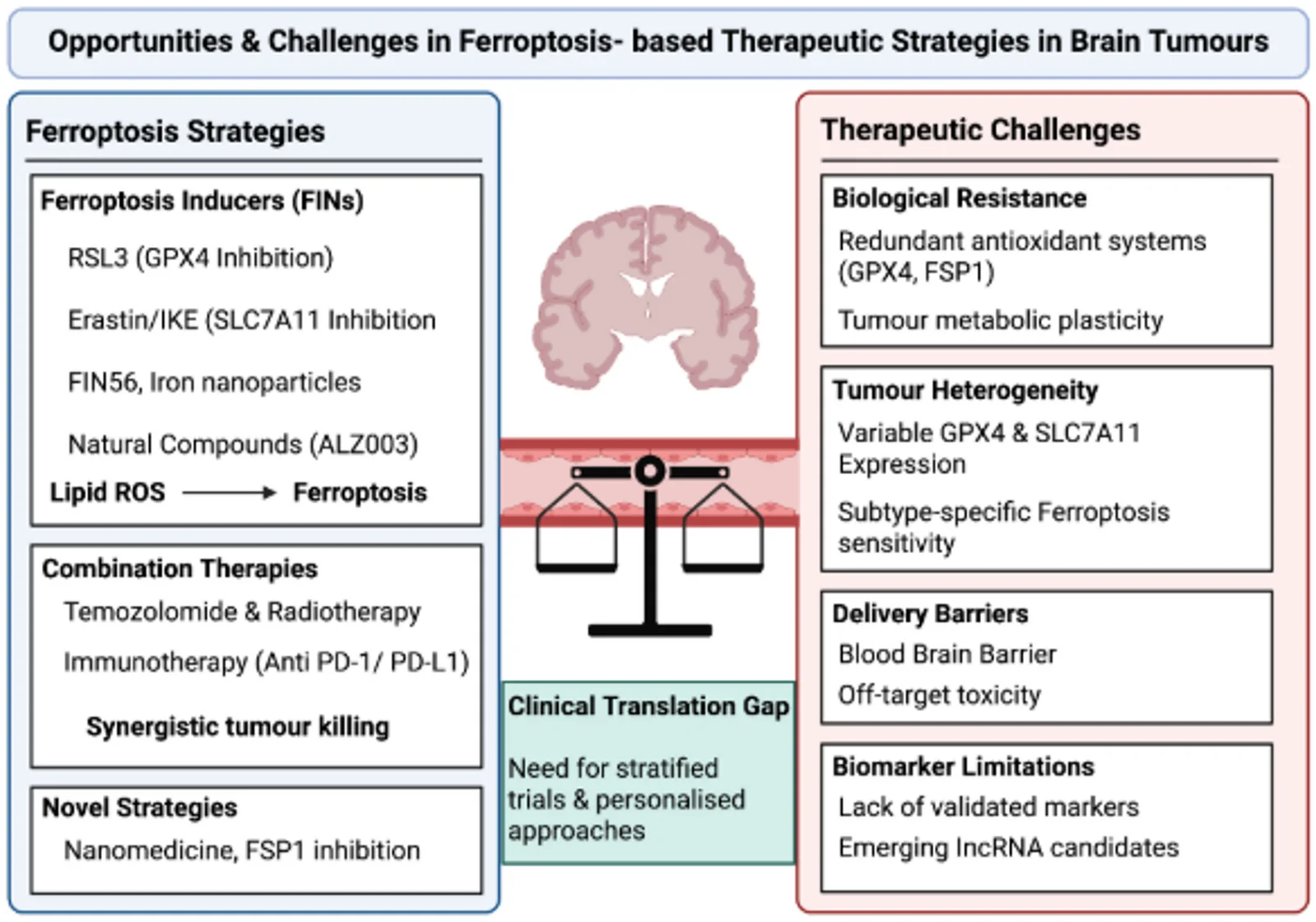
Opportunities and challenges in ferroptosis-based therapeutic strategies in brain tumours. Overview of ferroptosis-targeting approaches, including ferroptosis inducers (FINs), combination therapies, and emerging strategies such as nanomedicine and FSP1 inhibition. Key translational challenges are highlighted, including biological resistance, tumour heterogeneity, delivery barriers such as the blood–brain barrier, and limitations in biomarker development, contributing to the clinical translation gap

The prospect of targeting various metabolic pathways and genetic mutations to induce ferroptosis in brain tumours presents opportunities for personalised medicine. For instance, specific interventions targeting *GPX4*, *SLC7A11*, or iron metabolism can be tailored to individual patients based on tumour susceptibility and the presence of biomarkers indicative of a likely response to ferroptosis-based therapies [37]. These strategies, along with the therapeutic challenges, are depicted in Fig. 2 to demonstrate a visual comparison.

Despite the encouraging preclinical results the translational evidence supporting ferroptosis-targeted therapies remains subject to important limitations. Much of the mechanistic and therapeutic data originates from established glioblastoma cell lines, which may not accurately represent the marked genetic, metabolic, and phenotypic heterogeneity observed in patient tumours. Long-term culture adaptation can alter ferroptosis sensitivity and treatment responses, potentially limiting the clinical relevance of findings obtained in vitro. Similarly, although xenograft models provide valuable proof-of-concept evidence, they frequently employ immunodeficient hosts and ectopic tumour implantation, which incompletely recapitulate the complex immune, stromal, and metabolic interactions present in human glioblastoma. Consequently, therapeutic efficacy observed in xenografts may overestimate the effectiveness of ferroptosis-inducing strategies in patients. Human glioma slice cultures preserve aspects of tumour architecture and cellular heterogeneity; however, their short-term viability and inability to reproduce dynamic vascular, metabolic, and immune interactions limit their utility for predicting long-term therapeutic outcomes.

Additional challenges arise from studies relying on transcriptomic signatures and retrospective clinical datasets to infer ferroptosis susceptibility. Many proposed biomarkers are derived from mRNA expression profiles of ferroptosis-related genes and therefore represent indirect surrogate markers rather than direct measurements of ferroptotic activity. Such signatures cannot determine whether phospholipid lipid peroxidation, iron-dependent membrane damage, or ferroptotic cell death have actually occurred. Furthermore, transcriptomic analyses fail to capture critical post-translational determinants of ferroptosis sensitivity, including GPX4 enzymatic activity, protein stability, iron bioavailability, and lipid-remodelling dynamics. Retrospective datasets provide useful associations between ferroptosis-related signatures and clinical outcomes but remain vulnerable to confounding factors, treatment heterogeneity, and selection bias, precluding causal inference. Therefore, although ferroptosis-associated biomarkers and therapeutic targets show considerable promise, their clinical utility requires prospective validation using direct functional and biochemical measures of ferroptosis in patient-derived models and clinical trials (Table 2).

**Table 2:** Comparative overview of the major ferroptosis regulators discussed throughout this review, summarising their biological function, mechanistic role in ferroptosis, and clinical relevance in brain tumours

Regulator	Biological function mechanism	Role in ferroptosis	Clinical relevance in brain tumours
GPX4 (glutathione peroxidase 4)	Uses glutathione (GSH) as a cofactor to reduce lipid hydroperoxides, the terminal step of the system X_c_^−^–GSH–GPX4 antioxidant axis.	Principal suppressor of ferroptosis; its inhibition or loss is the classical trigger of ferroptotic death.	Overexpressed in glioblastoma versus healthy astrocytes and correlates with therapy resistance [32, 33]; paradoxically decreased in relapsed tumours alongside elevated ACSL4, suggesting a recurrence-associated vulnerability [33].
SLC7A11/xCT (system X_c_^−^ subunit)	Cystine/glutamate antiporter subunit that imports cystine for intracellular GSH synthesis.	Sustains the GSH pool that fuels GPX4; upregulation confers ferroptosis resistance.	Elevated xCT drives glioma growth, survival, and invasion, and correlates with temozolomide resistance in recurrent glioblastoma [34, 35].
ACSL4 (acyl-CoA synthetase long-chain family member 4)	Esterifies polyunsaturated fatty acids (arachidonic/adrenic acid) into membrane phospholipids, creating peroxidation-prone substrate.	Pro-ferroptotic; increases membrane susceptibility to lipid peroxidation.	Elevated in relapsed/recurrent glioblastoma alongside GPX4 loss, indicating a paradoxical increase in ferroptosis susceptibility despite treatment resistance [33].
FSP1 (ferroptosis suppressor protein 1)	Glutathione-independent radical-trapping antioxidant, acting in parallel to the GSH–GPX4 axis.	Suppresses ferroptosis independently of GPX4; provides a bypass route for antioxidant defence.	Pharmacological inhibition (iFSP1) sensitises glioblastoma cells to ferroptosis inducers, marking it as a candidate combination-therapy target [112].
Iron metabolism (TFR1/ferritin FTH1–FTL)	TFR1 mediates cellular iron import; ferritin (FTH1/FTL) sequesters iron; their balance sets the labile iron pool available for Fenton chemistry.	Net iron flux determines susceptibility: high TFR1 with low ferritin increases free iron and lipid peroxidation.	Glioblastoma cells and glioma stem cells show heightened iron dependency (“iron addiction”); tight regulation of iron uptake/export/storage is an acquired resistance mechanism [37, 38, 39].

## Discussions

### Key unanswered questions

Despite the rapid growth of ferroptosis research, several critical questions remain unresolved with regard to brain tumours. First, it is unclear whether recurrent tumours are universally more susceptible to ferroptosis or whether this vulnerability is limited to specific cellular states and anatomical niches. As noted in Sect. Preclinical and clinical observations primary–recurrent tumour comparison, glioblastoma recurrent samples have exhibited reduced *GPX4* and increased *ACSL4*, aligning with heightened ferroptotic susceptibility; however, other datasets suggest that tumour evolution simultaneously enhances antioxidant programmes and immune-suppressive circuits, potentially reducing the efficacy of ferroptosis induction. This indicates that recurrence should be viewed as a dynamic balance between new vulnerabilities and adaptive resistance, rather than a singular ferroptosis state [33, 41, 114].

Secondly, the role of ferroptosis in regulating antitumour immunity in vivo remains a subject of debate. On one hand, as detailed in Sect. Crosstalk with immune cells, CD8 + T cells can induce tumour ferroptosis via IFN-γ-mediated suppression of system X_c_^−^ components, linking adaptive immunity with lipid peroxidation in tumours [44]. This was further supported by Liao et al. [45], who showed that IFN-γ and arachidonic acid interact through *ACSL4* to promote immunogenic ferroptosis. Conversely, evidence indicates that ferroptotic cancer cells may hinder dendritic cell maturation and antigen presentation, suggesting that ferroptosis is not always immunostimulatory and may, in certain contexts, suppress immune responses [50]. In glioma, states enriched with ferroptosis have been linked to increased immunosuppression and macrophage infiltration, highlighting that ferroptosis can be either therapeutically advantageous or immunologically harmful, depending on factors such as timing, intensity, and cellular targets [115].

A further unresolved issue concerns selectivity: are immune cells inherently more vulnerable to ferroptotic injury than tumour cells? This question is especially pertinent in brain tumours, where effective anti-tumour immunity is hindered by limited T-cell infiltration, dominance of myeloid cells, and the blood-brain barrier. Current research considers ferroptosis at a compartmental level, recognising that tumour cells, dendritic cells, macrophages and cytotoxic lymphocytes each have their own thresholds. Practically, this implies that therapies inducing ferroptosis might fail if they damage antigen-presenting cells or exhausted but salvageable T cells more than tumour cells. This concern is heightened in glioblastoma, where immune function is already compromised and regional microenvironments vary markedly in oxygen levels, lipids, and oxidative stress [44, 50, 114].

Ultimately, the ability to spatially control ferroptosis remains uncertain. Glioblastoma is organised into distinct ecological and transcriptional niches, with malignant cells shifting among astrocyte-like, mesenchymal-like, neural progenitor-like and oligodendrocyte progenitor-like states influenced by genetic factors and the microenvironment [116]. Spatial transcriptomic studies support the idea that vulnerabilities are region-specific rather than uniform across the tumour, while recent work shows that dependence on *GPX4* is especially marked in certain quiescent astrocyte-like glioma populations [41, 117]. The key translational question, therefore, is less about whether glioblastoma is sensitive to ferroptosis, and more about which cells, in which niches, and at what stage they are most vulnerable.

### Future directions

#### Advancement of intelligent and targeted nano-delivery systems

A primary future focus is the development of brain-permeable, spatially selective delivery systems for therapeutics that induce ferroptosis. Nanomedicine is particularly promising in neuro-oncology because it can enhance crossing the BBB, improve tumour retention, enable controlled release, and reduce off-target neurotoxicity. Recent reviews of glioma nanoplatforms highlight that inorganic nanoparticles, biomimetic carriers, and polymer-based systems can augment reactive oxygen species, deplete glutathione, and facilitate local ferroptosis within glioma tissue [108]. More broadly, reviews on brain cancer nanomedicine also identify significant translational barriers, including neurotoxicity, uncertainties in biodistribution, and the need for tumour-specific surface ligands [118]. Future research should prioritise stimuli-responsive and lysosome-targeted nanocarriers while also considering pharmacokinetics, intracranial safety, and reproducible manufacturing.

#### Integration of spatial and single-cell technologies for precision mapping

Another key priority is the integration of spatial transcriptomics, single-cell sequencing, metabolomics, and imaging techniques to delineate ferroptosis-sensitive niches more precisely. Current glioblastoma models are often limited by bulk tissue averaging, which can obscure heterogeneity within tumours in redox status, immune microenvironment, and metabolic profile. Spatial and single-cell approaches now reveal that glioblastoma consists of organised cellular states, influenced by neighbouring vasculature, hypoxia, immune infiltrates, and the extracellular matrix [116, 117]. Future studies should move beyond descriptive mapping and directly overlay ferroptosis regulators such as GPX4, SLC7A11, ACSL4, FSP1, DHODH, and iron-handling genes onto spatially resolved tumour atlases. This would help identify whether hypoxic cores, invasive margins, perivascular niches, or therapy-resistant cell populations are the most tractable targets for ferroptosis-based intervention.

#### Personalised and biomarker-driven approaches

Translating research into clinical practice requires the development of more reliable biomarkers. Although ferroptosis-related gene signatures are increasingly reported in glioma, many remain retrospective, derived from transcriptome data, and insufficiently validated across different cohorts or tissue contexts. Biomarkers for future use should combine molecular, metabolic, and spatial information rather than rely on a single gene or pathway. Candidate methods include ferroptosis-related gene panels, lipid peroxidation markers, iron-handling profiles, and paired immune-context signatures that indicate when ferroptosis is likely to stimulate effective anti-tumour immunity rather than immune dysfunction. Evidence shows that reduced expression of system X_c_^−^ correlates with stronger CD8 and interferon-γ programmes during immunotherapy, while datasets in glioma suggest ferroptosis-related states may also be associated with malignancy and immunosuppression [44, 114, 115].

#### Interdisciplinary and AI-enabled innovation

The inherent complexity of ferroptosis in brain tumours makes this an interdisciplinary challenge. Progress depends on collaboration across neuro-oncology, immunology, lipid biochemistry, redox biology, nanotechnology, pathology, and computational science. Machine learning can help integrate multimodal data and reveal spatial patterns that are difficult to detect manually, such as niche-specific ferroptosis risks, treatment-induced metabolic shifts, and immune-ferroptosis feedback loops. This is especially relevant as glioblastoma is not a static mass but a dynamic ecosystem of transcriptional states, regional habitats, and treatment-adapted clones. AI-guided models could assist in patient stratification, predicting effective drug combinations, and identifying which tumour regions should be targeted first to maximise ferroptotic impact with minimal collateral effects [114, 116].

### Translational roadmap and clinical outlook

A pragmatic roadmap for clinical translation should progress through distinct stages. The initial phase involves refining mechanistic insights to identify which brain tumour types, cellular states, and microenvironmental niches rely most on *GPX4*, system X_c_^−^, *ACSL4*, *FSP1*, or mitochondrial pathways that confer protection against ferroptosis. The subsequent step is the qualification of biomarkers, ideally employing paired primary and recurrent specimens with detailed spatial annotations. The third phase focuses on optimising delivery methods, particularly for compounds or nanoplatforms designed to traverse the BBB and target infiltrative tumours. Until these steps are completed, confident progression into early-phase trials guided by specific biomarkers should be deferred. While promise exists, advancement remains hindered by the absence of approved ferroptosis-based cancer treatments and uncertainties regarding therapeutic windows, immune effects, and CNS-specific toxicity [114].

A significant and emerging concern is the risk of ‘on target off tumour’ toxicity within the central nervous system. Since ferroptosis results from iron-catalysed peroxidation of polyunsaturated membrane lipids, the brain’s unique physiology makes it particularly vulnerable to ferroptotic damage, due to its high oxygen consumption, abundant polyunsaturated fatty acids, and substantial regional iron deposition. Many ferroptosis-inducing agents, such as system xC and GPX4 inhibitors, target pathways vital to healthy neural cell survival. Without spatial or molecular targeting, such therapies could cause collateral damage to non-malignant tissues. Neurons, being post-mitotic and rich in lipids, are at risk of ferroptosis-related damage seen in ischemic, traumatic, and neurodegenerative conditions. Oligodendrocytes are especially vulnerable due to their high iron demand for myelination and the lipid-rich nature of myelin, which increases the risk of demyelination and white matter injury. Ferroptosis in brain microvascular endothelial cells can weaken tight junctions, impairing the blood-brain barrier and potentially worsening oedema and drug distribution issues. Microglia and infiltrating immune cells are also affected; notably, cytotoxic T cells and antigen-presenting cells are sensitive to ferroptosis. An inducer that preferentially depletes these immune cells over tumour cells would undermine the immune-metabolic rationale for ferroptosis therapy, as discussed by Li et al. [32] and Liu et al. ([55]. These issues underscore the importance of tumour-specific delivery, well-defined therapeutic windows, and preclinical models capable of detecting toxicity in neurons, glia, vasculature, and immune cells before moving to clinical trials.

In brain tumours, in particular, an effective trial design would likely require combination therapy rather than sole targeting of ferroptosis. Preclinical data support combining with radiotherapy, temozolomide, immune checkpoint blockade, or nano-enabled delivery systems, provided these combinations are tested with careful attention to timing and compartmentalisation. A likely clinical approach might begin with recurrent glioblastoma, where ferroptosis sensitivity may be increased and standard options limited, integrating serial tissue or liquid biomarkers to confirm target engagement. Such an approach would align mechanistic insights from recurrent glioblastoma samples and context-specific GPX4 dependence with the practical aim of de-risking initial CNS applications [33, 41, 108].

## Study limitations

This review possesses several limitations. Firstly, it is a narrative overview; although structured using SANRA, it lacks quantitative synthesis, reproducibility measures, or formal risk-of-bias assessments typical of systematic reviews or meta-analyses. Secondly, the evidence predominantly derives from preclinical studies, including cell lines, xenografts, organotypic cultures, and mechanistic reviews, with limited direct clinical data on patients with brain tumours. This imbalance is notable in ferroptosis research, where biochemical plausibility is high, but therapeutic application remains nascent; currently, no ferroptosis-based anticancer therapies have been approved [19, 114].

An additional limitation concerns heterogeneity in how ferroptosis is defined and assessed across studies. Some reports infer ferroptosis solely from transcriptional signatures, while others rely on lipid peroxidation assays, cellular morphology, rescue experiments with ferroptosis inhibitors, or the expression of regulators such as *GPX4*,* ACSL4*, and *SLC7A11*. Inferring ferroptosis from transcriptomic signatures constitutes weak evidence: gene expression levels serve only as an indirect proxy and do not measure lipid peroxidation flux or actual cell death. They also neglect post-translational regulation, enzyme activity, and the availability of polyunsaturated fatty acid substrates, all of which ultimately determine whether a cell undergoes ferroptosis. Consequently, such signatures establish correlation rather than direct evidence of active ferroptotic death. Many are derived from retrospective bulk transcriptomic cohorts, such as The Cancer Genome Atlas and Chinese Glioma Genome Atlas, which are susceptible to confounding factors like treatment effects and batch variability, lack spatial and single-cell resolution, and are prone to overfitting. Relatively few signatures have been validated prospectively or through functional studies across independent cohorts and tissue contexts. This methodological variability complicates comparisons between studies and challenges the identification of genuine ferroptotic processes versus broader oxidative stress responses. The issue is further magnified in glioblastoma, where regional heterogeneity, cellular plasticity, and immune composition mean that one tumour region may not reflect the biology of another [114, 116, 117]

A further translational issue relates to models of the central nervous system. Many ferroptosis investigations utilise models that inadequately replicate the BBB, neuroinflammatory responses, myeloid dominance, or treatment-induced spatial remodelling in recurrent disease. In addition, most mechanistic data derive from 2-dimensional cell lines, xenografts, or organotypic slice cultures, each of which distorts ferroptosis biology in specific and consequential ways rather than simply offering an incomplete picture. Established glioma cell lines pose significant challenges because serial passaging tends to select for genetic changes that diverge from the parental tumour [120, 121]. Culture in serum-supplemented media and exposure to atmospheric oxygen levels, which are approximately 21%, far above physiological brain oxygen tensions, directly influence membrane lipid composition, glutathione levels, and oxidative balance. These factors govern susceptibility to ferroptosis and thus confound the very phenotype under investigation [122]. Xenografts, particularly those established in immunodeficient hosts, lack a functional adaptive immune system and therefore cannot assess the immunometabolic interactions central to this review. Additionally, murine stroma, vasculature, and iron and lipid metabolism differ markedly from human systems [123]. Subcutaneous models also omit the integrity of the blood-brain barrier. Organotypic slice cultures maintain tissue architecture temporarily but are limited by tissue injury from sectioning, reactive gliosis, the progressive loss of microglia and perfused vasculature, and non-physiological oxygen levels outside the living organism. These limitations reduce their fidelity and restrict the window for viable observation [124]. Similarly, novel nanomedicine approaches are promising but remain largely proof-of-concept; many have yet to demonstrate long-term safety, standardised manufacturing processes, or effective intracranial delivery in humans [108, 118].

Finally, given the rapid development of this field, particularly regarding spatial omics, immune-ferroptosis interactions, and nanodelivery systems, some mechanistic interpretations must remain provisional. The present review aims not to establish a definitive model but to synthesise evolving literature into a clinically relevant framework centred on ferroptosis as an immunometabolic checkpoint, shaped by tumour spatial ecology.

## Conclusion

Ferroptosis has emerged as more than a non-apoptotic cell death pathway in brain tumours; it is increasingly recognised as an immunometabolic checkpoint situated at the intersection of iron regulation, lipid peroxidation, redox defence, and tumour-immune interactions. In glioblastoma, in particular, this framework helps explain why ferroptosis can be both therapeutically promising and biologically intricate. Tumour cells leverage antioxidant systems such as system xC − and *GPX4* to withstand oxidative stress, yet recurrent disease and specific cellular states may also expose vulnerabilities that can be targeted. Therefore, A key conclusion of this review is that understanding ferroptosis in brain tumours requires consideration of spatial heterogeneity. Glioblastoma is organised into microenvironmental niches and transcriptional states that differ in metabolism, immune exposure, oxygen levels, and stress responses. Accordingly, susceptibility to ferroptosis is unlikely to be uniform across the tumour. Future therapeutic advances will depend on identifying the most vulnerable tumour regions and delivering treatments with sufficient precision to exploit these weaknesses without inflicting unnecessary damage on healthy brain tissue or beneficial immune populations.

## Data Availability

No datasets were generated or analysed during the current study.

## Abbreviations

AA: Arachidonic acid
ACSL4: Acyl‑CoA synthetase long‑chain family Member 4
ALDH1A3: Aldehyde Dehydrogenase 1 Family Member A3
ALZ003: Curcumin analogue ALZ003
ATP: Adenosine triphosphate
BBB: Blood–brain barrier
BCL‑2: B‑cell Lymphoma 2
CD8 + T cells: Cluster of Differentiation 8–positive T Lymphocytes
CSF: Cerebrospinal fluid
DAMP: Damage‑associated molecular pattern
DMT1: Divalent metal transporter 1
DNA: Deoxyribonucleic acid
FIN: Ferroptosis inducer
FIN56: Ferroptosis‑inducing agent 56
FSP1: Ferroptosis suppressor Protein 1
FTH1: Ferritin heavy chain 1
FTL: Ferritin light chain
GPX4: Glutathione peroxidase 4
GSC: Glioma stem cell/glioma stem‑like cell
GSH: Glutathione
HIF: Hypoxia‑inducible factor
HMGB1: High‑mobility group box 1
HO1: Heme oxygenase‑1
HSPB1: Heat shock protein beta‑1
IDH: Isocitrate dehydrogenase
IFN‑γ: Interferon gamma
IKE: Imidazole ketone erastin
IL‑10: Interleukin‑10
MGMT: O6‑methylguanine‑DNA Methyltransferase
MLKL: Mixed‑lineage kinase domain‑like protein
MDA: Malondialdehyde
NF‑κB: Nuclear factor Kappa B
NRF2: Nuclear factor erythroid 2‑related factor 2
OTUB1: OTU Deubiquitinase, Ubiquitin Aldehyde‑binding 1
PD‑1: Programmed cell death Protein 1
PD‑L1: Programmed death‑ligand 1
PR: Progesterone receptor
PUFA: Polyunsaturated Fatty Acid
RAGE: Receptor for advanced glycation end product
ROS: Reactive oxygen species
RSL3: RAS‑selective Lethal 3
scRNA‑seq: Single‑cell RNA sequencing
SANRA: Scale for the assessment of narrative review articles
SHH: Sonic Hedgehog
siRNA: Small Interfering RNA
SLC3A2: Solute carrier family 3 Member 2
SLC7A11: Solute carrier family 7 Member 11
TGF‑β: Transforming growth factor beta
TFRC: Transferrin receptor 1
TME: Tumour microenvironment
U87MG: Human glioblastoma cell line U87MG
U251: Human glioblastoma cell line U251
xCT: Cystine/glutamate antiporter (SLC7A11)
